# Using Hepatic Gene Expression Assays in English Sole (*Parophrys vetulus*) to Investigate the Effects of Metro Vancouver Wastewater Effluents

**DOI:** 10.3390/toxics11080657

**Published:** 2023-07-29

**Authors:** Karan Parekh, Vicki L. Marlatt

**Affiliations:** Department of Biological Sciences, Simon Fraser University, Burnaby, BC V5A 1S6, Canada; kparekh@sfu.ca

**Keywords:** biomarkers, endocrine disrupter, differential expression, RT-qPCR, vitellogenin

## Abstract

The present study has investigated the effects of Metro Vancouver’s wastewater treatment plant (WWTP) effluents on English sole (*Parophrys vetulus*) hepatic gene expression using novel targeted gene expression assays to complement the 2017 Burrard Inlet Ambient Monitoring Program conducted by Metro Vancouver. Seven locations of varying distance to the WWTPs were included. Twelve genes involved in xenobiotic defense (*CYP1A*, *HSP70*), thyroid function (*DIO1*), lipid and glucose metabolism (*FABP1*, *FASN*, *GLUT2*, *PPAR*δ, *PPAR*γ), protein synthesis (*18S rRNA*, *RPS4X*), and reproduction (*ER*α, *VTG*) revealed several differences between these impacted sites. A key finding of the present study was that males exhibited VTG transcript levels either equivalent or exceeding female levels of this gene at all sites investigated, indicating widespread exposure of estrogenic contaminants throughout Burrard Inlet. Furthermore, the induction of hepatic CYP1A was observed due to possible downstream sites being subjected to a larger influx of certain planar halogenated and non-halogenated hydrocarbons from multiple industrial contributors. This study also revealed significant differences between the sites examined and in genes involved in transcriptional regulation and synthesis of proteins, lipids and glucose metabolism, and thyroid hormone metabolism. Collectively, this study demonstrates the potential of molecular biomarkers of urban contaminant exposure in wild caught English sole for use in diagnosing a broader range of adverse health effects when combined with conventional whole organism health indicators.

## 1. Introduction

Contaminants have been identified as one of Earth’s greatest threats to human and environmental health [1]. This is in large part a result of decades of industrial, agricultural, and urban anthropogenic activities deliberately or accidentally releasing numerous chemical pollutants into waterways [2]. Of particular concern are the hundreds of chemicals released from municipal wastewater treatment plants (WWTPs), which include contaminants such as metals, polycyclic aromatic hydrocarbons (PAHs), polychlorinated dibenzo-p-dioxins and polychlorinated dibenzofurans, surfactants (i.e., alkylphenol ethoxylates), pesticides, polybrominated diphenyl ethers (PBDEs), polychlorinated biphenyls (PCBs), human hormones, pharmaceuticals and personal care products (PPCPs; i.e., prescription, over the counter, and veterinary therapeutic drugs for human and animal ailments/diseases, and PCPs such as soaps, deodorants, and cosmetics) [3]. Despite the known adverse effects of several of these chemicals on aquatic wildlife, toxicity data is lacking for many chemicals in WWTP effluents. Furthermore, the toxic effects of various chemical mixtures that reflect environmentally relevant exposure scenarios near WWTP discharge sites complicate risk assessments.

Pharmaceuticals discharged in WWTP effluents are of particular concern since they have been designed to target various processes in humans that are often conserved across vertebrate species; thus, many are toxic to aquatic wildlife at low concentrations (ng/L to µg/L) alone or as mixtures (i.e., additive, or synergistic) [4]. Indeed, despite the known adverse effects of several PPCPs on aquatic wildlife, a limited number of ambient environmental quality guidelines exist to assess the risks of PPCPs to aquatic wildlife that are currently used in many countries. For example, in Canada, nonylphenol, nonylphenol ethoxylates, and 17α-ethynylestradiol (EE2) currently have working water and sediment quality guidelines for the protection of aquatic life [5,6]. Whereas in Europe, priority pollutant lists under the European Water Framework Directive (EU WFD) include ambient quality guidelines for estrogenic compounds of biological (e.g., 17β-estradiol (E2), estrone (E1), estriol (E3)) and synthetic nature (e.g., bisphenol A (BPA), octylphenol (OP), nonyohenol (NP), and EE2) [7]. The ambient quality guidelines for estrogenic compounds have arisen due to the irrefutable data on their ability to disrupt the endocrine system and in particular, mimic estrogen [8,9]. Research on the impacts of a low-level synthetic birth control estrogen, EE2, on fish revealed compelling connections between molecular and population-level effects [8]. Specifically, chronic waterborne exposure to EE2 induced vitellogenin (VTG), gonadal abnormalities, and ultimately led to the near extinction of the species in an experimental lake [8]. Furthermore, elevated concentrations of BPA (a plasticizer) and OP (surfactant) in wastewater effluents have been shown to be associated with feminisation of male fish, amphibians, and reptiles [9]. Indeed, close to an additional 800 chemicals are known to be capable of interfering with endocrine systems and have been deemed endocrine disrupting compounds (EDCs) [10]. Specifically, an EDC has been defined as an “exogenous substance or mixture that alters function(s) of the endocrine system, and consequently, causes adverse health effects in an intact organism, or its progeny, or (sub)populations” [10]. Interestingly, despite the few environmental quality guidelines and regulations for the hundreds of chemicals, including EDCs, discharged via WWTPs, there is consensus within the scientific community that contaminants in WWTP effluents are causing adverse effects in downstream aquatic wildlife populations [11].

In Metro Vancouver, there are five WWTPs, with two of primary and three of secondary treatment sewage processing. To evaluate the potential adverse impacts of effluents from these WWTPs on aquatic wildlife, regular and comprehensive field monitoring programs are conducted every five years. These programs assess the concentrations of contaminants in water, sediment, and biota. One specific program, the Burrard Inlet Ambient Monitoring Program (BIAMP), focusses on Burrard Inlet and Iona Island to examine various contaminants and their effects on fish and their habitat proximal to two primary WWTPs, Lions Gate and Iona [12,13,14].

The objective of this study was to complement the most recent BIAMP conducted by Metro Vancouver in 2017 by evaluating the effects of WWTP effluents on English sole (*Parophrys vetulus*) hepatic gene expression profiles for several target genes, and identify associations to whole body contaminant concentrations and various health indices [12]. The approach was to harness the robust molecular biomarkers of contaminant exposures (i.e., VTG and cytochrome P450) in fish and develop additional potential biomarkers involved in a variety of biological processes using English sole, a species of relevance to the west coast of North America. English sole is an important benthic flatfish in commercial, tribal, and recreational fisheries, and has ecological contributions to shelf habitats [15]. With a widespread distribution from Baja California, Mexico to the Aleutian Islands, Alaska, the English sole possess a distinct advantage for monitoring the health of marine ecosystems across a vast geographical area [15,16]. Notably, these fish exhibit a high degree of site fidelity, remaining in niche areas throughout their lives, enabling the assessment and identification of spatial patterns of contamination related to urbanization or other point source pollutants over time [17]. Therefore, to accomplish this objective, several novel RT-qPCR assays were developed and used to assess hepatic gene expression in wild female and male English sole collected from various locations in Burrard Inlet and near and far-field from the Iona Deep-Sea Outfall. The genes of interest investigated were designed to evaluate adverse effects on various biological processes and/or systems, including xenobiotic detoxification, thyroid hormone metabolism, lipid and glucose metabolism, and reproduction.

## 2. Materials and Methods

### 2.1. Sampling of Wild English Sole

**Figure 1:**
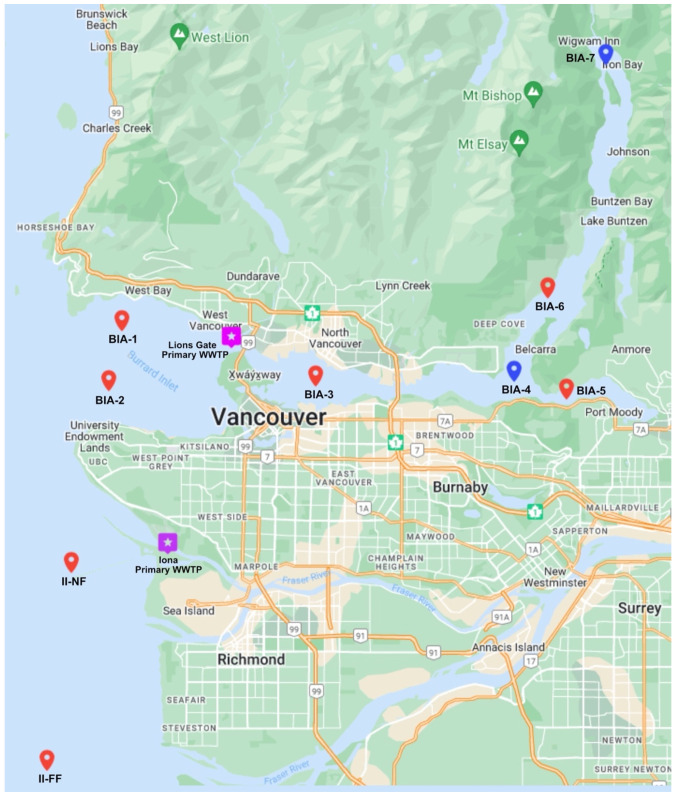
Location of seven sampling sites for the BIAMP involved in water, sediment, and English sole collections [12,13,14]. In 2017, the BIAMP included five sites along the Burrard Inlet (BIA-1, BIA-2, BIA-3, BIA-5, BIA-6), two sites associated with Iona Island (II-NF, II-FF), and two of Metro Vancouver’s primary WWTPs, Lions Gate and Iona (in purple). The map highlights current sampling sites in red and historical sites in blue, with the omission of one site at the mouth of the Indian Arm (BIA-4) and a reference site at the North end of the Indian Arm (BIA-7).

The present study utilized BIAMP data, collected by ENKON Environmental and Metro Vancouver, to obtain wild English sole from seven sites (BIA-1; BIA-2; BIA-3; BIA-5; BIA-6; II-NF; and II-FF) conducted during September 2017 (Table 1 and Figure 1) [12]. Each site was sampled for fish communities, and a minimum of three bottom trawls were conducted using a scientific otter trawl net. The number of fish collected at each site ranged between 17 and 22, and only mature English soles were retained for further analyses [12]. The fish were sacrificed via blunt force trauma, and organs were extracted for sex identification and measurement of health indices and morphometrics (i.e., fork length (mm), whole body weight (g), liver weight (g), and gonad weight (g)) [12]. Liver tissues were collected, snap frozen, and stored at −80 °C for long-term subsequent analyses. All work with English soles was performed in compliance with the guidelines of the Canadian Council for Animal Care and with a permit issued by Metro Vancouver (Vancouver, BC, Canada).

### 2.2. RNA Isolation and cDNA Synthesis

Approximately 100 mg of individual English sole liver samples were homogenized with 1 mL TRIzol^®^ (Invitrogen, Burlington, ON, Canada) and 1 mm tungsten-carbide beads for 4 min at 30 Hz using the Retsch Mixer Mill MM400 instrument (Fisher Scientific, Ottawa, ON, Canada). Total RNA was extracted following the manufacturer’s instructions, with TURBO DNA-free kits^TM^ (Ambion, Austin, TX, USA) used to remove genomic DNA contamination. RNA quality was assessed using the Bio-Rad Experion^TM^ Automated Electrophoresis System (Version 3.20; Bio-Rad, Mississauga, ON, Canada) with an average RNA quality indicator (RQI) score ± standard error of the mean of 8.35 ± 0.18 for 136 biological samples. RNA concentrations were quantified using an Epoch 2 Microplate Spectrophotometer (BioTek, Winooski, VT, USA), with a mean 260/280 nm absorbance ratio of 2.11 ± 0.08, indicating high purity RNA. cDNA was synthesized using the Applied Biosystems High-Capacity cDNA Synthesis Kit (Waltham, MA, USA) according to the manufacturer’s instructions, and quantified using the Epoch 2 Microplate Spectrophotometer. The resulting cDNA products were diluted in double-distilled water to obtain final concentrations of 100 ng/μL prior to use in RT-qPCR reactions.

### 2.3. Primer Design

Gene-specific primer sets for RT-qPCR assays were designed based on highly similar phylogenetic ancestries in mitochondrial 12S and 16S sequences, assessed via BLAST (https://www.ncbi.nlm.nih.gov/, accessed on 1 November 2020), from the European plaice (*Pleuronectes platessa*), European flounder (*Platichthys flesus*), olive flounder (*Paralichthys olivaceus*), and the Pacific halibut (*Hippoglossus stenolepis*) [18]. Primers were designed using the Clustal Omega Multiple Sequence Alignment tool, and screened for optimal melting temperatures (T_M_) and guanine-cytosine content (GC%) via the Integrated DNA Technologies OligoAnalyzer^TM^ Tool (www.idtdna.com/calc/analyzer, accessed on 1 January 2021). All gene-specific primer sets were PCR-amplified according to the manufacturer’s instructions using the Bio-Rad T100 Thermal Cycler (Mississauga, ON, Canada) and ABM Taq DNA Polymerase (Applied Biological Materials, Richmond, BC, Canada), and verified through Sanger Sequencing at the University of British Columbia (UBC) Sequencing and Bioinformatics Consortium (Vancouver, BC, Canada). Primer sequences, corresponding amplicon sizes, and PCR efficiencies are listed in Table 2.

### 2.4. Real-Time Quantitative Polymerase Chain Reaction

RT-qPCR gene amplification was conducted on the Bio-Rad CFX384^TM^ Real-Time PCR Detection System (Bio-Rad, Mississauga, ON, Canada) using 2.5 µL of diluted cDNA template (1:80 dilution, 100 ng/µL); 0.25–0.375 µL each of the forward and reverse gene-specific primers (10 µM); 6.25 µL of the SsoFast^TM^ EvaGreen^®^ Supermix (Bio-Rad, Mississauga, ON, Canada), and nuclease-free water to complete the 12.5 µL reaction in a 384-well plate. The thermocycling profile included an initial denaturation step at 95 °C, for 30 s, followed by 45 amplification cycles consisting of 5 s at 95 °C for denaturation, 5 s at 55 °C for primer annealing, and 15 s at 72 °C for extension (fluorescence data was collected during the extension step). To check the specificity of the reaction and the presence of primer dimers, a dissociation protocol with a gradient from 55 °C to 95 °C was employed. Each plate included two technical replicates for each biological sample and a no-template control, as well as a positive plate control. RT-qPCR efficiency calculations were performed using seven-point standard curves of a 4-fold dilution series (1:4–1:16′384) from pooled cDNA in each assay.

### 2.5. Data Analysis of Gene Expression

The Livak ΔΔCq method was used to determine relative quantitation of target genes between exposure sites and reference genes. Reference gene stability was evaluated by calculating M-values using the Bio-Rad CFX Manager^TM^ Software (Mississauga, ON, Canada). Two reference genes with M-values < 1 were used to normalize the expression of target genes and compensate for differences in sample tissue amount [19].

### 2.6. Statistical Analyses

Statistical analyses were performed using JMP^®^, Ver. 16 (SAS Institute Inc., Cary, NC, USA). Normality and homogeneity of variance for each normalized gene expression dataset were tested using Shapiro-Wilk’s and Levene’s tests, respectively. Logarithmic transformation was applied to normalized gene expression data that failed normality tests. Outliers were identified and removed if individual observations were scored greater than ±3 based on studentized residuals. Two-Way ANOVA followed by a Tukey’s post-hoc analysis was used to determine statistical significance of the least square means within and between sites, sexes, and the interactions between these two variables. The significance level was set at a *p*-value < 0.05.

## 3. Results

### 3.1. Wild English Sole Health and Body Morphometrics

**Figure 2:**
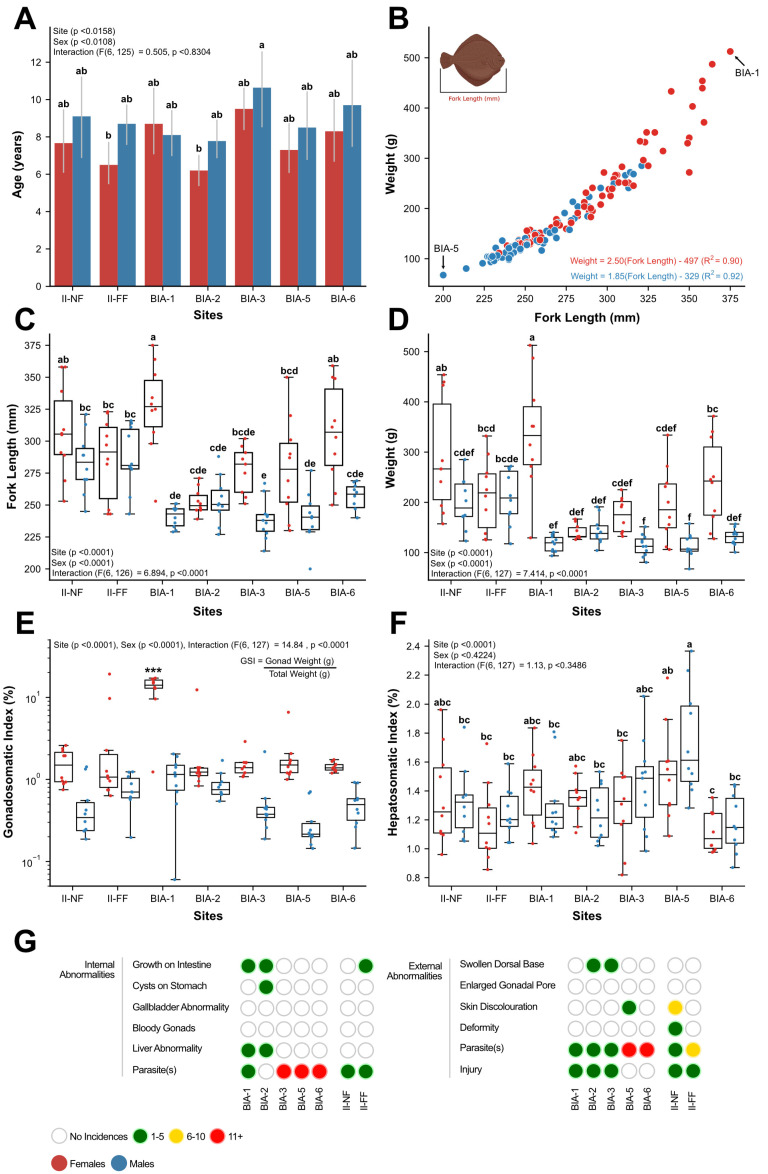
Various fish heath measures in English sole collected during the 2017 BIAMP [12] at seven sites: (**A**) Mean age ± one standard error of the mean; (**B**) Relationship between fork length and wet body weigh; (**C**) Fork length; (**D**) Wet body weight; (**E**) Gonadosomatic index; (**F**) Hepatosomatic index; and (**G**) Internal and external abnormalities in males and females pooled within each site. Boxplots show median values, 25th and 75th percentiles, minimum and maximum values, and outliers. A two-way ANOVA followed by a Tukey’s post-hoc test was used to determine significant differences between main effects (site and sex) and their interaction. Different superscripts indicate significant differences (*p* < 0.05).

In the present study, we examined a population of mature English sole, encompassing males aged between 5 and 16 years, and females aged between 4 and 16 years [12]. Analysis of the data revealed variations in the age structure among the sampling sites (*p* < 0.0158) and sexes (*p* < 0.0108), but no interaction between the main effects was observed (F(6, 125) = 0.505, *p* < 0.8304) [12]. The oldest individuals were found at BIA-3, with a mean age of 11.4 years for males and 9.5 years for females, whereas the youngest individuals were observed at BIA-2, with a mean age of 7.8 years for males and 6.2 years for females (Figure 2A) [12]. Furthermore, growth rates exhibited sex-related differences, with females showing faster growth compared to males (Figure 2B) [12]. Notably, the largest and heaviest female English sole was found at the Outer Harbour (BIA-1), near the Lion’s Gate WWTP discharge site, while no such pattern was observed in males (Figure 2C,D) [12]. Additionally, a significant disparity in the levels of the gonadosomatic index (GSI) among the various collection sites was observed (*p* < 0.0001; Figure 2E) [12]. Female English sole from BIA-1 exhibited remarkably higher GSI levels, averaging around 13%, in comparison to all other sites where GSI levels ranged ~1.3–3.7% (*p* < 0.0001) [12]. Additionally, a noteworthy discovery was the consistency of GSI values among males compared to females across all sites [12]. Lastly, the hepatosomatic index (HSI) levels showed similar levels in both males and females (*p* < 0.4224), with the highest HSI levels being recorded at BIA-5 and the lowest at BIA-6 (*p* < 0.0001; Figure 2F) [12].

### 3.2. Gene Primer Set Design and Evaluation

A total of 14 primer sets used to amplify candidate genes of interest generated single amplicons, as demonstrated by the presence of single bands in agarose gel electrophoresis, single-peak melting curves of the PCR products, and via third-party verification through Sanger Sequencing at the UBC Sequencing and Bioinformatics Consortium (Table 2). A full list of all unsuccessful genes of interest including data related to primer design as well as detailed reasons for exclusion are provided in Table S1. Efficiency of RT-qPCR reactions ranged 90–110% with one minor exception to these criteria, the case of *HSP70*, which exhibited an efficiency of 112% but was deemed acceptable due to the presence of a single sharp melt peak and R^2^ = 0.971. The M-value for the two reference genes, *ACT*β and *eEF1*α*1*, was 0.58 with an average coefficient variance (CV) of 0.20.

### 3.3. RT-qPCR Gene Expression

**Figure 3:**
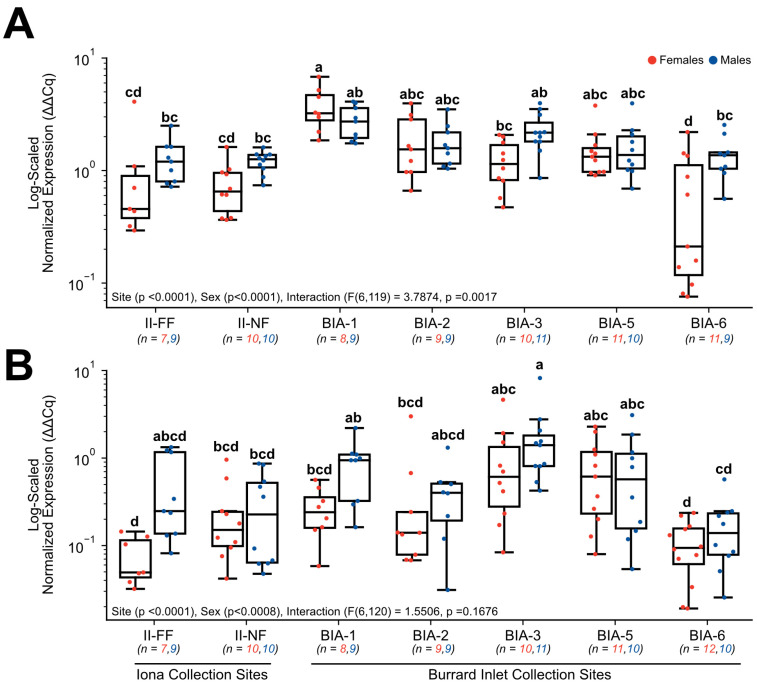
Hepatic gene expression levels of (**A**) *18S rRNA* and (**B**) *RPS4X* in English sole at near- and far-field sites from the Iona and Lion’s Gate WWTP in Burrard Inlet. Boxplots show median values, 25th and 75th percentiles, minimum and maximum values, and outliers. Two-Way ANOVA with Tukey’s post-hoc analysis was used to determine significant differences between main effects (site and sex) and their interaction. Biological replicates for each site are provided. Significant site and sex effects were observed in both genes, and a significant interaction effect was observed for 18S rRNA. Different superscript letters indicate significant differences (*p* < 0.05).

Mean normalized expression (± standard error of the mean) values of 7–11 biological replicates per site per sex for a total of 12 genes of biological interest revealed several significant differences between sites and sexes, and in some instances, the interactive effects between these two main factors. Target genes involved in protein synthesis, which included *18S rRNA* (Figure 3A) and *RPS4X* (Figure 3B), both exhibited significant changes in normalized gene expression. Normalized expression in *18S rRNA* was found to have a main effect of site (*p* < 0.0001), sex (*p* < 0.0001), and the interaction (F(6, 119) = 3.7874, *p* = 0.0017). Specifically, normalized expression was significantly elevated in females at BIA-1 (3.75 ± 0.58) in comparison to some sites further from the Lion’s Gate WWTP (BIA-3 (1.23 ± 0.18), BIA-6 (0.66 ± 0.22)) and compared to both Iona WWTP sites investigated (II-NF = 0.76 ± 0.12, II-FF = 1.06 ± 0.52; Figure 3A). However, changes in *18S rRNA* appeared to be sex-specific since no significant changes were observed for males between sites (*p* > 0.05; Figure 3A). In addition, differences in the expression of *18S rRNA* were significant between sexes at one site, whereby BIA-6 females (0.66 ± 0.22) exhibited significantly lower normalized expression of *18S rRNA* compared to males (1.39 ± 0.20) at this site (Figure 3A). Normalized expression in *RPS4X* was found to have a main effect of site (*p* < 0.0001) and sex (*p* < 0.0008), but no significant interaction was observed (F(6, 120) = 1.5506, *p* = 0.1676). Specifically in males, normalized expression was significantly elevated at BIA-3 (1.90 ± 0.66) in comparison to BIA-6 (0.18 ± 0.05) and to the near-field Iona WWTP site (II-NF = 0.34 ± 0.10); whereas females at BIA-6 revealed significantly lower normalized expression of *RPS4X* in comparison to BIA-5 (0.83 ± 0.23) and BIA-3 (1.10 ± 0.44; Figure 3B). Interestingly, at both Iona WWTP sites, males and females exhibited similar levels of *RPS4X* that were not significantly different from any of the Lion’s Gate WWTP sites, except for females at II-FF (0.08 ± 0.02), which differed from several sites (BIA-3 = 1.10 ± 0.44, BIA-5 = 0.83 ± 0.23; Figure 3B).

**Figure 4:**
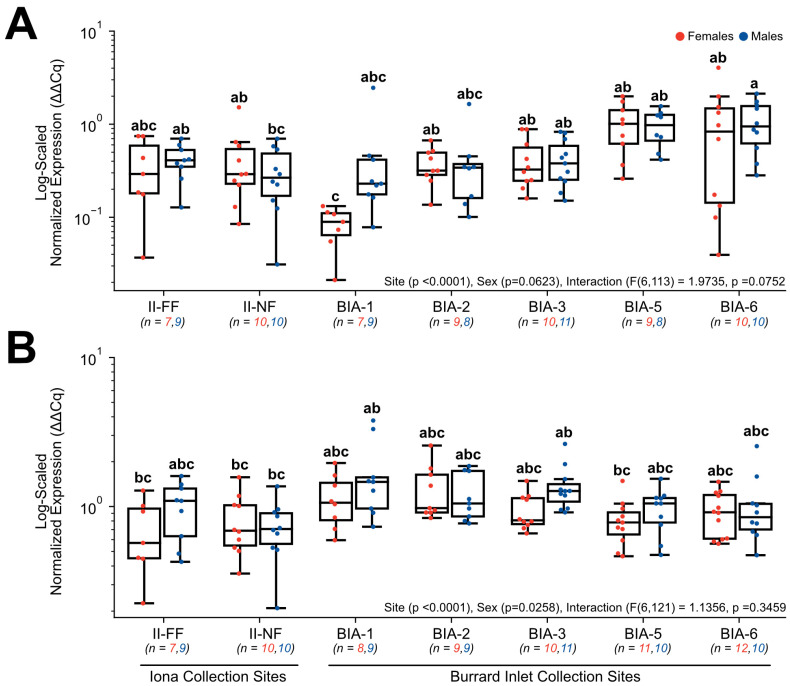
Hepatic gene expression levels of (**A**) *CYP1A* and (**B**) *HSP70* in English sole at near- and far-field sites from the Iona and Lion’s Gate WWTP in Burrard Inlet. Boxplots show median values, 25th and 75th percentiles, minimum and maximum values, and outliers. Two-Way ANOVA with Tukey’s post-hoc analysis was used to determine significant differences between main effects (site and sex) and their interaction. Biological replicates for each site are provided. Significant site effects were observed in both genes, while *HSP70* showed a significant sex effect. However, no interactive effect was found. Different superscript letters indicate significant differences (*p* < 0.05).

Target genes involved in xenobiotic detoxification and transcriptional regulation, which included *CYP1A* (Figure 4A) and *HSP70* (Figure 4B), both exhibited significant changes in normalized gene expression. For *CYP1A*, there was no significant effect of sex (*p* = 0.0623) or interactive effects between sex and site (F(6, 113) = 1.9735, *p* = 0.0752), but site as a main effect was significant (*p* < 0.0001). Within females, normalized expression was significantly lower nearest to the Lion’s Gate WWTP at BIA-1 (0.08 ± 0.01) in comparison to sites further from the Lion’s Gate WWTP (BIA-2 = 0.38 ±0.05, BIA-3 = 0.43 ± 0.09, BIA-5 = 1.03 ± 0.20, BIA-6 = 1.10 ± 0.39), and compared to the near-field Iona WWTP site (II-NF = 0.44 ± 0.13; Figure 4A). However, within males, no significant differences were observed across Lion’s Gate and Iona WWTP sites, but BIA-6 (1.09 ± 0.20) exhibited higher *CYP1A* expression relative to II-NF (0.32 ± 0.07; Figure 4A). Normalized expression in *HSP70* was found to be a main effect of site (*p* < 0.0001) and sex (*p* = 0.0258) but no significant interaction was observed (F(6, 121) = 1.1356, *p* = 0.3459; Figure 4B).

**Figure 5:**
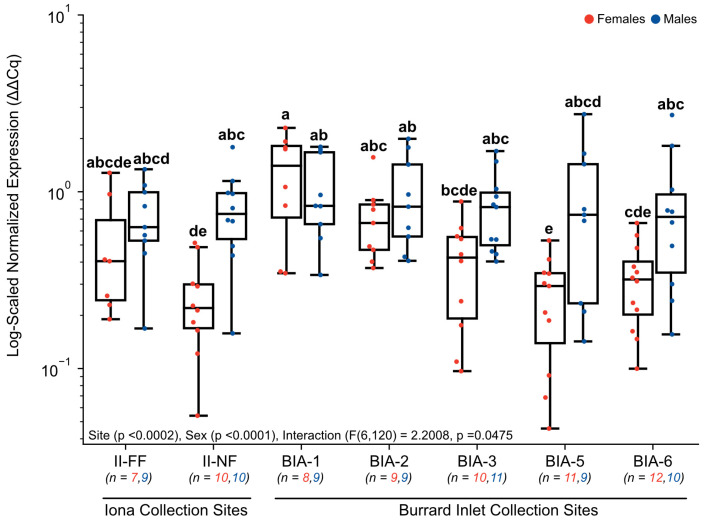
Hepatic gene expression levels of *DIO1* in English sole at near- and far-field sites from the Iona and Lion’s Gate WWTP in Burrard Inlet. Boxplots show median values, 25th and 75th percentiles, minimum and maximum values, and outliers. Two-Way ANOVA with Tukey’s post-hoc analysis was used to determine significant differences between main effects (site and sex) and their interaction. Biological replicates for each site are provided. A significant effect of site, sex, and an interactive effect was observed. Different superscript letters indicate significant differences (*p* < 0.05).

Target genes involved in homeostasis and metabolic function like *DIO1* exhibited significant changes in normalized gene expression (Figure 5). Normalized expression in *DIO1* was found to have a main effect of site (*p* < 0.0002), sex (*p* < 0.0001), and the interaction (F(6, 120) = 2.2008, *p* = 0.0475). Specifically, normalized expression was significantly elevated in females at BIA-1 (1.29 ± 0.26) in comparison to some sites further from the Lion’s Gate WWTP (BIA-3 (0.41 ± 0.08), BIA-5 (0.26 ± 0.05), BIA-6 (0.33 ± 0.05)), and compared to one of the Iona WWTP sites, II-NF (0.26 ± 0.05; Figure 5). However, changes in *DIO1* appeared to be sex-specific since no significant changes were observed for males between sites (*p* > 0.05). In addition, differences in the expression of *DIO1* were significant between sexes at two sites, whereby BIA-5 (0.26 ± 0.05) and II-NF (0.26 ± 0.05) females exhibited significantly lower normalized expression of *DIO1* compared to males (BIA-5 = 0.96 ± 0.28, II-NF = 0.82 ± 0.14; Figure 5) at these sites.

**Figure 6:**
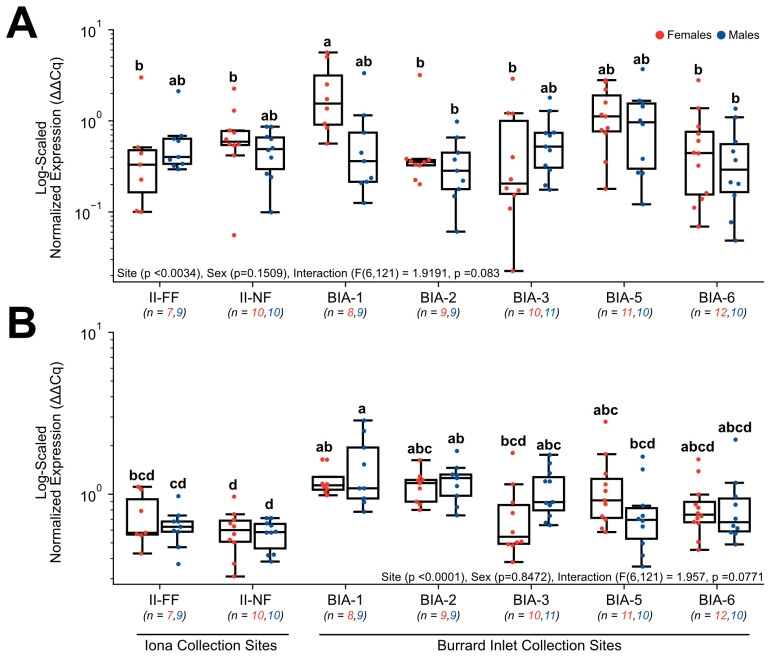
Hepatic gene expression levels of (**A**) *FABP1* and (**B**) *FASN* in English sole at near- and far-field sites from the Iona and Lion’s Gate WWTP in Burrard Inlet. Boxplots show median values, 25th and 75th percentiles, minimum and maximum values, and outliers. Two-Way ANOVA with Tukey’s post-hoc analysis was used to determine significant differences between main effects (site and sex) and their interaction. Biological replicates for each site are provided. Significant site effects were observed in both genes; however, there were no significant effects for sex or the interaction in either gene. Different superscript letters indicate significant differences (*p* < 0.05).

**Figure 7:**
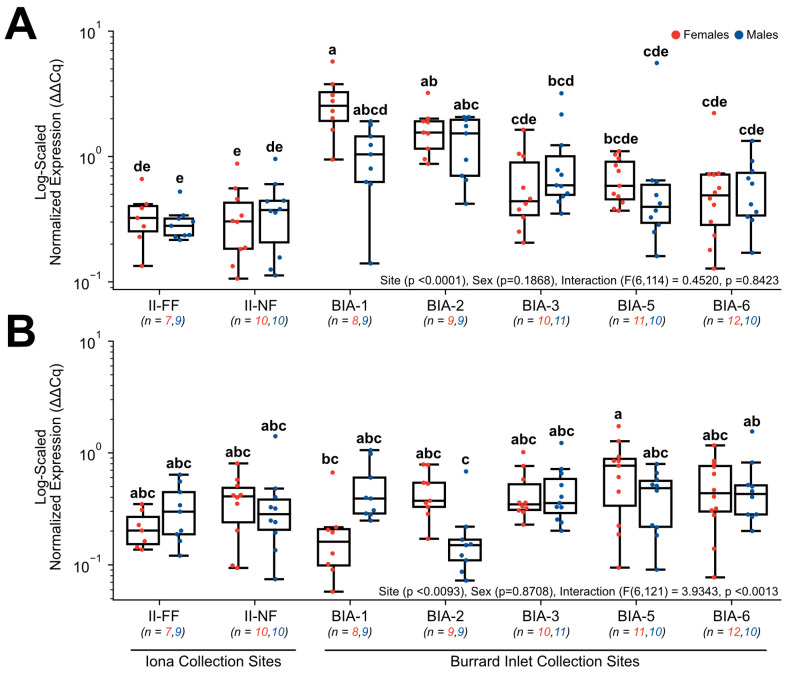
Hepatic gene expression levels of (**A**) *PPAR*δ and (**B**) *PPAR*γ in English sole at near- and far-field sites from the Iona and Lion’s Gate WWTP in Burrard Inlet. Boxplots show median values, 25th and 75th percentiles, minimum and maximum values, and outliers. Two-Way ANOVA with Tukey’s post-hoc analysis was used to determine significant differences between main effects (site and sex) and their interaction. Biological replicates for each site are provided. Significant site effects were observed in both genes, with the interactive effect specifically observed in *PPAR*γ. However, neither gene showed a main effect of sex. Different superscript letters indicate significant differences (*p* < 0.05).

Target genes involved in lipid and glucose metabolism included *FABP1* (Figure 6A), *FASN* (Figure 6B), *PPARδ* (Figure 7A), and *PPAR*γ (Figure 7B), and all exhibited significant changes in normalized gene expression. For *FABP1*, there was no significant main effect of sex (*p* = 0.1509) or interactive effects between sex and site (F(6, 121) = 1.9191, *p* = 0.083), but site as a main effect was significant (*p* < 0.0034). Within females, normalized expression was significantly higher nearest to Lion’s Gate WWTP at BIA-1 (2.33 ± 0.69) in comparison to sites further from the Lion’s Gate WWTP (BIA-2 = 0.64 ± 0.96, BIA-3 = 0.66 ± 0.29, BIA-6 = 0.67 ± 0.22) and both Iona WWTP sites (II-NF = 0.78 ± 0.19, II-FF = 0.67 ± 0.39, Figure 6A). However, changes in *FABP1* appeared to be sex-specific since no significant changes were observed for males between sites (*p* > 0.05). For *FASN*, there was no significant main effect of sex (*p* = 0.8472) or interactive effects between sex and site (F(6, 121) = 1.957, *p* = 0.0771), but site as a main effect was significant (*p* < 0.0001). Within males, normalized expression was significantly higher nearest to Lion’s Gate WWTP at BIA-1 in comparison to BIA-5 (0.8 ± 0.14) and compared to both Iona WWTP sites investigated (II-NF = 0.57 ± 0.04, II-FF = 0.64 ± 0.06, Figure 6B). Interestingly, females exhibited similar levels of *FASN* that were not significantly different from any of the Lion’s Gate WWTP sites, except BIA-1 (1.22 ± 0.09), which exhibited significantly higher normalized expression of *FASN* compared to Iona WWTP sites (II-NF = 0.60 ± 0.06, II-FF = 0.73 ± 0.11, Figure 6B). Normalized expression in *PPAR*δ was found to have a main effect of site (*p* < 0.0001), but no significant main effect of sex (*p* = 0.1868) and the interaction (F(6,114) = 0.4520, *p* = 0.8423) was observed. Within females, normalized expression was significantly higher nearest to Lion’s Gate WWTP at BIA-1 (2.78 ± 0.52) in comparison to some sites further from the primary WWTP (BIA-3 = 0.63 ± 0.14, BIA-5 = 0.68 ± 0.08, BIA-6 = 0.60 ± 0.16), and compared to both Iona WWTP sites investigated (II-NF = 0.34 ± 0.07, II-FF = 0.35 ± 0.06; Figure 7A). However, within males, the far-field Iona WWTP site (II-FF = 0.30 ± 0.03) exhibited lower *PPAR*δ expression relative to several sites at Lion’s Gate WWTP (BIA-1 = 1.07 ± 0.20, BIA-2 = 1.34 ± 0.22, BIA-3 = 1.00 ± 0.27), whereas the near-field Iona WWTP site (II-NF = 0.39 ± 0.08) only exhibited lower *PPAR*δ expression relative to BIA-2 (1.34 ± 0.22; Figure 7A). For *PPAR*γ, there was no significant main effect of sex (*p* = 0.8708), but site as a main effect (*p* < 0.0093) and the interactive effects between these two main factors (F(6, 121) = 3.9343, *p* < 0.0013) were significant. Specifically, normalized expression was significantly elevated in females at BIA-5 (0.72 ± 0.15) in comparison to the nearest site to the Lion’s Gate WWTP (BIA-1 = 0.21 ± 0.07; Figure 7B). Similarly, normalized expression was significantly elevated in males at BIA-6 (0.53 ± 0.13) in comparison to the BIA-2 (0.20 ± 0.06). Lastly, normalized expression in *GLUT2* indicated no main effect of site (*p* = 0.0751), sex (*p* = 0.4418), nor an interactive effect between these two main factors (F(6, 114) = 0.4520, *p* = 0.8423; data not shown).

**Figure 8:**
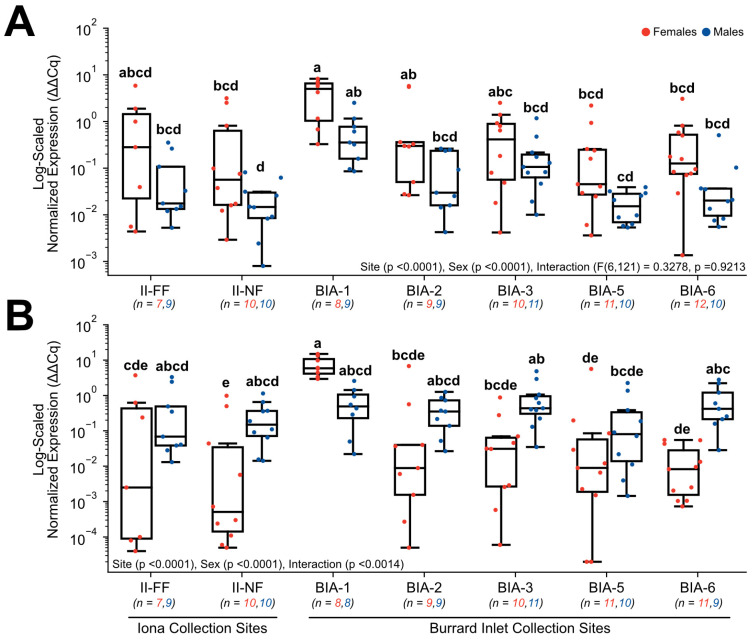
Hepatic gene expression levels of (**A**) *ERα* and (**B**) *VTG* in English sole at near- and far-field sites from the Iona and Lion’s Gate WWTP in Burrard Inlet. Boxplots show median values, 25th and 75th percentiles, minimum and maximum values, and outliers. Two-Way ANOVA with Tukey’s post-hoc analysis was used to determine significant differences between main effects (site and sex) and their interaction. Biological replicates for each site are provided. Significant site and sex effects were observed in both genes, with the interactive effect specifically observed in *VTG*.

Target genes involved in reproduction included *ER*α (Figure 8A) and *VTG* (Figure 8B), and both exhibited significant changes in normalized gene expression. Normalized expression in *ER*α was found to have a main effect of site (*p* < 0.0001) and sex (*p* < 0.0001), but no interactive effects between these two main factors were observed (F(6,121) = 0.3278, *p* = 0.9213). Within females, normalized expression was significantly higher nearest to Lion’s Gate WWTP at BIA-1 (4.23 ± 1.10) in comparison to some sites further from the primary WWTP (BIA-5 = 0.36 ± 0.20, BIA-6 = 0.47 ± 0.25) and compared to the near-field Iona WWTP site (II-NF = 0.67 ± 0.37; Figure 8A). Similarly, within males, normalized expression was also significantly higher nearest to Lion’s Gate WWTP at BIA-1 (0.67 ± 0.26) in comparison to BIA-5 (0.02 ± 0.004) and compared to the near-field Iona WWTP site (II-NF = 0.03 ± 0.008; Figure 8A). Normalized expression in *VTG* was found to have a main effect of site (*p* < 0.0001), sex (*p* < 0.0001), and the interaction (*p* < 0.0014). Specifically, normalized expression was significantly elevated in females nearest to Lion’s Gate WWTP at BIA-1 (7.58 ± 1.63) in comparison to all sites further from the primary WWTP (BIA-2 = 0.83 ± 0.75, BIA-3 = 0.13 ± 0.09, BIA-5 = 0.54 ± 0.51, BIA-6 = 0.02 ± 0.01) and compared to both Iona WWTP sites investigated (II-NF = 0.15 ± 0.10, II-FF = 0.65 ± 0.52; Figure 8B). However, changes in *VTG* appeared to be sex-specific since no significant changes were observed for males between sites (*p* > 0.05). In addition, differences in the expression of *VTG* were significant between sexes at two sites, whereby BIA-6 (0.88 ± 0.33) and II-NF (0.30 ± 0.11) males exhibited significantly higher normalized expression of *VTG* compared to females (BIA-6 = 0.02 ± 0.007, II-NF = 0.15 ± 0.10; Figure 8B) at these sites.

## 4. Discussion

### 4.1. Examining the Health and Body Metrics of Resident English Sole

Several studies have successfully demonstrated the application of the GSI to improve accuracy and precision in determining sexual maturity and fertility in fish [20,21], and collectively this study demonstrates the potential of molecular biomarkers of urban contaminant exposure in wild caught English sole for use in diagnosing a broader range of adverse health effects when combined with conventional whole organism health indicators (summarized in Table S2). For instance, the heavier body weight of females at BIA-1 was likely partially attributed to the significantly higher GSI in these females, the latter indicating ovaries in an advanced stage of development and closer to a ripe ovary observed just prior to spawning. With all English sole of adult age, one would expect the reproductive status to be similar across all sites; thus, it is possible that WWTP contaminants, particularly those with estrogenic modes of action, may have caused advanced ovarian development in females residing nearest the Lions Gate WWTP effluent discharge site (BIA-1). Although no detailed studies of English sole reproductive cycles and gonad size in BC have been conducted, English sole gonad size has been shown to vary seasonally in other Pacific Northwest populations. Specifically, GSI and gonad sizes increase during the months leading up to winter spawning, at which point GSI and gonad sizes peak, and this is then followed by reduced size immediately post-spawning of spent gonads and continued gonadal recrudescence into the summer and/or early fall [22]. In the present study, we found that female English sole collected closest to the Lion’s Gate WWTP (near BIA-1) exhibited markedly higher GSI levels resembling those of pre-spawning females. However, fish collected from all other sites exhibited GSIs that resembled those of spawned-out females. This advanced ovarian development at BIA-1 may be due to estrogenic contaminants known to be present in WWTP effluent (i.e., 17β-estradiol, estriol, estrogen 17α-ethynyl estradiol, etc.), and all other sites were exhibiting underdeveloped ovaries. Despite the evident differences in female GSI, critical lifelong ecological patterns of English sole in the Burrard Inlet related to spawning, planktonic life stages, settlement, and foraging have remained largely unexplored. As a result, the intriguing advanced ovarian development observed in these fish could potentially be attributed to the unique reproductive ecology specific to the English sole in this region of the Burrard Inlet, coupled with the presence of estrogenic contaminants stemming from the nearby WWTP. Therefore, to avoid unfounded assumptions, further in-depth research is imperative to establish a comprehensive understanding of the reproductive ecology of English sole in the area.

### 4.2. Influence of Urban Pollutants on Hepatic CYP1A Gene Expression

The cytochrome P450 detoxification system is an extensively studied enzyme system that is involved in the first phase oxidation reactions of the two-phase metabolism of xenobiotics and its activity, transcript, and protein abundance has been shown to be related to sediment, whole-body, and liver tissue concentrations of various planar halogenated and non-halogenated hydrocarbons [23]; and the present study showed significant differences in the expression levels of *CYP1A* transcripts in female English sole between sites of interest. Indeed, several studies over the past two decades have demonstrated the induction of hepatic *CYP1A* enzymes or transcripts after exposure to several industrial compounds, including PCBs, PBDEs, OCPs, DDT, and PAHs, primarily through the binding and activation of the AhR pathway [24]. For instance, in a 2000 field study, results found that sediment PCBs and PAHs near the Orange County wastewater discharge (California, USA) significantly increased CYP1A protein levels in English sole, with sediment PCBs showing a dose-dependent positive correlation [25]. Similarly, high molecular weight PAHs (e.g., naphthalene, fluorene, anthracene, pyrene) have been commonly associated with petroleum operations, lumber production, WWTPs, and commercial use throughout the Burrard Inlet, and while the BIAMP aims to monitor and assess the impacts of effluents from the primary Lion’s Gate WWTP, 2017 tissue chemistry results for arsenic, lead, PCBs, OCPs (represented by total DDT and total chlordane), dioxins and furans, and the PAH metabolite, pyrene 1-glucuronide in bile, were highest in fish from BIA-5 and BIA-6 [12]. It is possible that this is due to the Port Moody and Indian Arm being subjected to a larger influx of PAHs from multiple contributors, such as Imperial Oil Ioco, Flavelle Cedar, Burrard Thermal, and active hydroelectric plants relative to the Outer Harbour, in which two sampling sites (BIA-1 and BIA-2) were set [12,13,14]. Furthermore, liver English sole CYP1A activity measured using an in vitro EROD assay was significantly higher in the eastern portions of Burrard Inlet (BIA-5, BIA-6) compared to the western Outer Harbour region (BIA-1) in 2007, 2012, and 2017 [12,13,14]. These results suggest the exposure of English sole to CYP1A-inducing chemicals (e.g., dioxins and furans, PCBs, PAHs, and 2,3,7,8-TCDF) in Metro Vancouver waters generally supports the hypothesis that sites furthest from the Lion’s Gate WWTP had higher levels of these chemicals due to local contaminant sources in the Port Moody Arm (near BIA-5) and Indian Arm (near BIA-6) [12,13,14]. Nevertheless, PCBs and PBDEs have been measured at concentrations equal to or exceeding Canadian Environmental Quality Guidelines in Metro Vancouver’s WWTP, but are also prevalent from a variety of other sources in urban areas relative to both industrial and urban wastewater discharges [12,13,14].

### 4.3. Exploring Sewage Effluent’s Potential Disruption of Thyroid Hormone Metabolism

Interestingly, over the past ~three decades there is mounting evidence that in teleosts, peripheral thyroxine (T_4_) deiodination to the biologically active triiodothyronine (T_3_) hormone or inactivated reverse T_3_ (rT_3_) or 3,3′-diiodothyronine (T_2_) via deiodinase enzymatic activity are known to be influenced by contaminants detected in wastewater effluents (e.g., plasticizers, flame retardants, surfactants, and pharmaceuticals) [26,27]. Specifically, the DIO1 enzyme is involved in both T_4_-outer ring deiodination (ORD) and T_4_-inner ring deiodination (IRD) catalytic reactions to produce active T_3_ and inactive rT_3_ hormones, respectively, whereas DIO2 and DIO3 participate solely in ORD and IRD reactions, respectively [27]. Despite, the lack of gene-specific biomarkers of contaminants that disrupt thyroid hormone metabolism, recent field-based studies in fish demonstrated the impacts on the abundance of deiodinase enzyme levels in waters downstream of WWTP discharge sites [28,29]. Considering several suspected and known EDCs with modes of action that disrupt the HPT endocrine axis of vertebrates, the findings in the present study and the presence of these contaminants in Metro Vancouver sewage effluents (i.e., alkylphenols, dioxins and furans, metals, PAHs, PBDEs, PCBs, pesticides, and PPCPs), it is likely that English sole are experiencing some disruption of the HPT axis [12,13,14].

### 4.4. Exploring Fatty acid Metabolism Transcripts as Promising Biomarkers of Sewage Effluent Exposure

In teleost, lipids play a major role in the storage and provision of energy through lipogenesis pathways occurring in the cytosol via *FASN*, a multienzyme complex which catalyzes the conversion of acetyl-CoA and malonyl-CoA to 16- and 18-carbon fatty acids (palmitic and stearic acids, respectively); while intracellular transport and metabolism of dietary lipids and exogenous lipophilic compounds are catalyzed by cytoplasmic FABP1 [30]. Nevertheless, nuclear receptors (i.e., peroxisome proliferator-activated receptors, PPARs) are key to these processes, and bind a broad range of ligands (e.g., fatty acids and fatty acid derivatives) and serve as major transcriptional regulators of enzymes involved in fatty acid metabolism and transport [30]. Together, in the present study, a trend of elevations in two enzymes and a transcriptional regulator (PPARδ) involved in lipid metabolism at the site most proximal to a primary WWTP effluent discharge site (BIA-1) coupled with the significantly heavier and longer females at this site provides evidence of altered metabolism in fish exposed to WWTP effluent. Whether these larger fish exhibited different levels of lipids or other metabolites or organ level alterations in metabolism was not measured in the study, but should be considered in future monitoring programs.

Although several contaminants discharged in primary and secondary WWTP effluents have been detected globally, a recent review of the two main types of lipid-regulating agents, statins, and fibrates reported that these cardiovascular drugs are the most consumed pharmaceuticals worldwide, and are routinely detected in the ng/L to µg/L range in global surface waters [31,32,33]. Although not measured in the Metro Vancouver BIAMP, it is expected that these biologically active pharmaceuticals targeting lipid metabolism in vertebrates are present to some extent proximal to Metro Vancouver’s WWTP. Furthermore, the alterations in transcripts related to lipid metabolism in the present study support this hypothesis. Even though most studies reporting significant effects of fibrates and statins on lipid metabolism are laboratory-based and variable, some WWTP effluent field-based exposures do exist, and demonstrate effects on lipids and lipid metabolites [34]. Such studies suggest the potential use of PPARs and lipid-metabolism-related transcripts as biomarkers for environmental contaminant risk assessment in fish; however, further studies are warranted.

### 4.5. Disruption of the Reproductive Endocrine Axis by Estrogenic Contaminant Exposure

Strong evidence of exposure to contaminants with an estrogenic mode of action was observed in this study based on male English sole *VTG* transcript levels either equivalent or exceeding female levels of this gene at all sites investigated. In oviparous vertebrates and some invertebrates (i.e., bivalves), the glycolipophosphoprotein VTG is the precursor of vitellin, which is used as the primary nutritional source for the developing embryo [35]. It is produced in the liver and regulated by estrogens and their receptors (ligand activated transcription factors) that mediate estrogen action, and then exported into the blood and sequestered in oocytes [35]. Although males possess the genes to produce VTG, this naturally occurs at much lower levels than in females. As a result, VTG at the transcript and protein levels has been validated as a reliable and robust biomarker of exposure to estrogenic substances [36,37,38,39]. In addition, most teleosts investigated to date exhibit sex-specific differences in ERα levels with females exhibiting higher levels than males [40]; however, this was not observed in the present study. Thus, these similar levels of ERα in males and females further support the hypothesis of the widespread presence of estrogenic contaminants in Metro Vancouver waters. Interestingly, at site BIA-1 nearest to the primary treatment Lion’s Gate WWTP, where females exhibited advanced ovarian development and elevated ERα levels, the males collected from this site exhibited the largest mean magnitude VTG transcript induction relative to males from other study sites. Previous Metro Vancouver BIAMPs conducted in 2007 and 2012 measured protein levels of VTG and reported elevated levels in males nearest to the Lion’s Gate WWTP discharge site in both years, and some induction in the Central Harbour (BIA-3), Port Moody Arm (BIA-5), and Indian Arm (BIA-6; n = 5 males per site) [13,14]. Although no true reference site was included in the present study, Johnson et al. [41] demonstrated a low frequency of detecting protein VTG in Puget Sound English sole at three non-urban sites (0–6%) compared to urban impacted sites (12–47%). Locally, in controlled laboratory-based studies on Metro Vancouver WWTP effluents, Osachoff et al. [42] reported significant induction of *VTG* mRNA transcript and plasma protein levels in juvenile rainbow trout (*Oncorhynchus mykiss*) after exposure to both Metro Vancouver WWTP influent and effluent, indicating the presence of estrogenic contaminants discharged from primary and secondary WWTPs in Metro Vancouver. Collectively, multiple years of monitoring programs suggest that English sole males are exhibiting elevated VTG, indicative of the widespread occurrence of estrogenic substances in Metro Vancouver waters, and females spawning nearest the Lion’s Gate WWTP discharge site may occur abnormally early due to advanced ovarian development. With clear evidence of organismal and population level impacts due to the disruption of the reproductive endocrine axis in fish downstream of sewage effluent discharge sites collected since the 1990s (reviewed in Marlatt et al., [43]), mitigation measures such as improved WWTP treatment processes (i.e., tertiary level) are the ideal solution.

## 5. Conclusions

Field studies, such as the BIAMP conducted by Metro Vancouver, provide invaluable information about point-source environmental pollution associated with WWTPs as well as other anthropogenic pollutants and stressors in urban waters. Here, we report the induction of well characterized biomarkers of reproductive endocrine axis abnormalities (i.e., elevated *VTG* and *ER*α in males and females, advanced ovarian development) that were most prevalent nearest the primary treatment Lion’s Gate WWTP, but ultimately, the gene expression analyses indicate widespread exposure to estrogenic contaminants throughout the Burrard Inlet. Routine VTG and sex steroid receptor assays are recommended for future monitoring in Burrard Inlet to track the spatial and temporal extent of WWTP effluent exposure and impacts on aquatic wildlife. Although few other genes in fish are as well characterized as *VTG* and *ER*α, several genes involved in lipid and glucose metabolism and one enzyme involved in thyroid hormone metabolism were also associated with exposure to primary WWTP effluent. We hypothesize the former is at least partly attributed to the lipid regulating pharmaceuticals known to be present near sewage discharge sites, while the latter potential thyroid hormone metabolism disruption was due to several chemicals present in WWTP and other industrial effluents (i.e., alkylphenols, dioxins and furans, metals, PAHs, PBDEs, PCBs, pesticides, and PPCPs), several of which were present in English sole tissue in the 2017 BIAMP [12]. This study demonstrates the high potential and applicability of molecular biomarkers of urban contaminant exposure in wild caught English sole to assess a wider range of adverse health effects when combined with conventional whole organism health indicators.

## Figures and Tables

**Table 1:** Sampling sites for English sole from Lions Gate and Iona primary WWTPs. Sites were designated by the BIAMP [12,13,14] and include five sites along the Burrard Inlet and two sites along Iona Island. For each site of interest, location coordinates (Universal Transverse Mercator, UTM) and brief site descriptions are provided.

Site	Location	Coordinates (UTM)	Description
BIA-1	Outer Harbour North	E 483752 N 5464073	1.5 km offshore to the south of Pacific Institute
BIA-2	Outer Harbour South	E 482935 N 5460679	1.5 km north of Spanish Bank
BIA-3	Inner Harbour	E 493841 N 5460963	West of Loch Katrine Bank
BIA-5	Port Moody Arm	E 506788 N 5459875	East of Port Moody Narrows
BIA-6	Indian Arm	E 505549 N 5464824	Representative southern site
II-NF	Iona Island	E 478523 N 5453266	2–4 km north of Iona diffuser
II-FF	Iona Island	E 477283 N 5442000	8–10 km of south Iona diffuser

**Table 2:** Primer sequences designed to amplify genes of interest to examine the effects of wastewater effluents in English sole through RT-qPCR. For each target gene of interest, the National Center for Biotechnology and Information accession identifiers, forward and reverse primer sequences (5′ → 3′), annealing temperatures (Tm), amplicon sizes, efficiency of primer pairs (%E), and goodness of fit of linear regression for the relative standard curves (R^2^) are provided for reference genes and target genes of interest.

Target Gene	Accession ID	Primer Sequence	Tm (°C)	Amplicon Size	Efficiency (%)	R^2^
Reference Housekeeping Genes						
*18S rRNA*	**XR_004613416.1**	F: GGTCTGTGATGCCCTTAGATG R: GCTTATGACCCGCGCTTAC	55.8	210	104.5	0.972
*ACTβ*	**XM_035181811.1**	F: GACCAACTGGGATGACATGG R: GCGTACAGGGACAGCACAGC	61	204	103	0.972
Genes of Biological Interest						
*CYP1A*	**AJ310693.1**	F: TGTGAGGACAGGAAGCTGGA R: GCTCCAAACAGGTCGTTGACA	58	86	96.9	0.992
*DIO1*	**AB362421.1**	F: ACAGATGGTTGGGCCTTCAC R: TGACTTTCCCAGCCTGAAGC	57.7	196	108.1	0.992
*eEF1* α *1*	**XM_035620145.1**	F: AAGATCCACATCAACATCGTG R: CAAACTTCCACAGAGCGATG	56.7	229	98.8	0.998
*ER* α	**XM_035143352.1**	F: GCTGAGGGATTTGAGATGGCT R: ATGTAGTCATTGTGACCCTGGATG	56.9	143	106.1	0.96
*FABP1*	**XM_020099114.1**	F: GAAGGTCAAGGCGGTGGTTC R: ACATGCGTTTGCTCGTCCTC	58.4	155	108.4	0.989
*FASN*	**XM_035162863.1**	F: GCAACGGCAATGACAAAGAGC R: TTTGTCTGGTTTCCGTGCCA	57.6	143	107.8	0.952
*GLUT2*	**AY521663.1**	F: CCGCGCTACCTCTACATCGT R: TGCTGCCTGTAGACGGAAGA	58.6	182	102.6	0.969
*HSP70*	**AF187726.1**	F: CAGTGCCCGCCTACTTCAAT R: TTCTGACCCAACCTTCTTGTCC	57.3	143	111.7	0.971
*PPAR* δ	**XM_035158970.1**	F: GACCTCGCTCCACCCTTTAC R: TCCAAGCCCGAATGTGGAAC	57.7	157	98.5	0.988
*PPAR* γ	**AJ243956.2**	F: TGTCAGTCACGCTCTGCTGAA R: TAGGAGATCAGGGTCCCGTCT	58.8	176	102.4	0.988
*RPS4X*	**XM_034569090.1**	F: GTTTGATACTGCCAACCTGTGC R: TTGGAGAGCCTGGTAGCGAA	57.5	150	99.7	0.976
*VTG*	**XM_035177204.1**	F: CAAGAGCCAGAGTTCACACA R: TGCAACAGCATAAGTCTCAAC	57.3	143	103.6	0.971

## Data Availability

Data available upon request.

## Supplementary Materials

The following supporting information can be downloaded at: https://www.mdpi.com/article/10.3390/toxics11080657/s1, Table S1: List of primer sequences designed to amplify genes of interest in English Sole that were unsuccessful for RT-qPCR experiments, and the reasons for exclusion; Table S2: Overview of molecular biomarker and RT-qPCR results of urban contaminant exposure in wild English sole.
