# Supplementary material for: Using Hepatic Gene Expression Assays in English Sole (Parophrys vetulus) to Investigate the Effects of Metro Vancouver Wastewater Effluents
Source: Toxics. 2023 Jul 29;11(8):657. doi: 10.3390/toxics11080657 (PMC10459354; doi:10.3390/toxics11080657)
Supplement: Supplementary file 1 [file toxics-11-00657-s001.zip › toxics-2472222-supplementary.pdf]

Table S1. List of primer sequences designed to amplify genes of interest in English Sole that were unsuccessful for RT-qPCR experiments, and the reasons for exclusion. For each target gene of interest, the National Center for Biotechnology and Information (https://www.ncbi.nlm.nih.gov/) accession identifiers, forward and reverse primer sequences (5' → 3') and amplicon sizes are provided.

Target Gene	Accession ID	Primer Sequence	Amplicon Size	Reason For Exclusion
AhR	XM_020106318.1	F: TTCCTCCACGGTCAGAGCAG<br>R: CCAGCTTGTGCTTGGTCCTG	151	>1 Peak Melt Curve<br>Efficiency > 110%<br>Failed Standard Curve
GST	X63761.1	F: AAGTCAGCCGAGGTGATGAGC<br>R: CAGGCGGCGTAGGATTCATTC	92	>1 Peak Melt Curve
IL-1β	AJ010640.1	F: CGAGTGGAGGACAAGACCATG<br>R: GCTCGGATGTGCTGATGTACC	145	>1 Peak Melt Curve<br>Efficiency > 110%<br>Failed Standard Curve
IL-6	GU985454.1	F: GGCTTGTCTCCACAGGTTGG<br>R:CAGACAAGTTCAGGGCTCACTC	197	>1 Peak Melt Curve<br>Efficiency > 110%<br>Failed Standard Curve
MMP23B	XM_020089187.1	F:TCATAAACGCCAGTGACACAG<br>R: CCGTGATGCCGTCGAAACA	186	Efficiency > 110%<br>Failed Standard Curve
PYGL	XM_035167960.1	F: AGCTGGAGGAGATGGAGGAG<br>R: CAAGAAACACGCTGCCAGTC	71	Failed Sanger Sequencing
SOD1	AJ291980.1	F: AGATGCTCACCCTCAATGGC<br>R:TTCCAAGATCGTCAGCTTTCTC	78	>1 Peak Melt Curve
SOD2	XM_020106318.1	F: TTCCTCCACGGTCAGAGCAG<br>R: CCAGCTTGTGCTTGGTCCTG	151	>1 Peak Melt Curve<br>Efficiency > 110%<br>Failed Standard Curve
SOD3	XM_020100342.1	F: CCAGAGGTCATGCTGCA<br>R: GGTGGCAATACATTCTTTGCTC	85	>1 Peak Melt Curve<br>Efficiency > 110%<br>Failed Standard Curve
SOCS2	XM_034578888.1	F: ATCAGACGAGAGCCGCATCG<br>R: GGCGGAGATGGTGAACAGGT	169	>1 Peak Melt Curve<br>Efficiency > 110%<br>Failed Standard Curve
THRα	D16461.1	F: CCGCCTCATTGTCCTGTGATT<br>R: CCCTGGAACAGAGACGCTAAG	187	>1 Peak Melt Curve<br>Efficiency > 110%<br>Failed Standard Curve
THRβ	D45245.1	F: GCCAAGCGGAAGCTAATCGA<br>R: ATGCGGATGAGGTCCCACTC	113	>1 Peak Melt Curve
TNFα	AB040448.1	F: ATTCACTCAGGGCGGCTTC<br>R: TCCGTGAAGAGCCATACCCT	183	>1 Peak Melt Curve<br>Efficiency > 110%<br>Failed Standard Curve
UBQ	XM_034582323.1	F: CTGGAGGATGGACGCACACT<br>R: CGCAGACGCAGAACCAGATG	74	>1 Peak Melt Curve
WAP65	KC521544.1	F: TCATCACATCGCTCACATCGTT<br>R:AAGAGGGAAATCGTCTGTTCGT	99	Poor Efficiency (> 110%) /<br>Low Expression (< 4 Points in Standard Curve)

Table S2. Overview of molecular biomarker and RT-qPCR results of urban contaminant exposure in wild English sole. For each site of interest, known point sources of contamination are provided with whole organism health indicators (e.g., EROD activity; external and internal abnormalities; fork length; GSI; plasma VTG; and weight) from 2007, 2012, and 2017 Burrard Inlet Ambient Monitoring Program reports[1,2,3]. Gene expression change patterns in males and females observed in the present study are summarized as increased (upward arrow) or decreased (downward arrow) relative to its respective site.

Site	Known Point Sources of Contamination	BIAMP Results	Gene Expression Changes[4]
BIA-1<br>(Outer Harbour North)	Lions Gate Primary WWTP	↓ EROD Activity[1,2,3]<br>↑ Fork Length &Weight[1,2,3]<br>↑ GSI[1,3]<br>↑ Plasma VTG[1]<br>↓ Plasma VTG[2]	↑ 18S rRNA<br>↓ CYP1A<br>↑ DIO1<br>↑ ERα<br>↑ FABP1<br>↑ FASN<br>↑ PPARδ<br>↑ VTG
BIA-2<br>(Outer Harbour South)	Lions Gate Primary WWTP	↓ EROD Activity[1,2]<br>↑ GSI[2]<br>↑ Plasma VTG[1]<br>↓ Plasma VTG[2]	↑ FASN<br>↑ PPARδ
BIA-3<br>(Inner Harbour)	Shipping Activities & CSOs<br>(e.g., Vancouver Wharves, BC Sugar, Neptune Bulk Terminal, Canada Harbour Place)	↑ External & Internal Abnormalities[2]	↑ RPS4X
BIA-5<br>(Port Moody Arm)	Industrial Sources<br>(e.g., Imperial Oil Ioco, Flavelle Cedar, Burrard Thermal)	↑ EROD Activity[1,2,3]<br>↑ External & Internal Abnormalities[2,3]<br>↑ Liver Lesions[1,2,3]<br>↑ Plasma VTG[1,2]	↑ CYP1A<br>↓ DIO1<br>↑ FABP1
BIA-6<br>(Indian Arm)	Hydroelectric Plants<br>(e.g., Buntzen Plants)	↑ EROD Activity[2,3]<br>↑ External & Internal Abnormalities[1,3]<br>↑ Liver Lesions[1,2,3]	↓ 18S rRNA<br>↑ CYP1A<br>↓ RPS4X
II-NF<br>(Iona Near-Field)	Iona Island Primary WWTP	N/A	↓ FASN<br>↓ PPARδ
II-FF<br>(Iona Far-Field)	Downstream of South Arm of Fraser River	N/A	↓ FASN<br>↓ PPARδ

[1] 2007 Metro Vancouver Ambient Burrard Inlet Monitoring Program Fish Health Survey Report [23]
[2] 2012 Burrard Inlet Ambient Monitoring Program [24]
[3] 2017 Burrard Inlet Ambient Monitoring Program [22]
[4] Present study
